# The complete plastome of *Spondias dulcis* (Anacardiaceae): an edible deciduous tree species from South America

**DOI:** 10.1080/23802359.2022.2126288

**Published:** 2022-10-03

**Authors:** Xu-Long Yang, Xin-Xin Xu, Hua-Feng Wang

**Affiliations:** Sanya Nanfan Research Institute, Hainan University, Sanya, China

**Keywords:** Tropical fruits, genetics, Hainan, breeding, systematics, plastome

## Abstract

*Spondias dulcis* is a deciduous tree in the family Anacardiaceae. The species originates in South America and now is widely cultivated in tropical areas due to its edible fruits. In this study, we find that the length of the complete plastome of *S. dulcis* is 162,256 bp. It includes 35 tRNA genes and eight rRNA genes, 86 protein-coding genes and totally 130 genes. The complete plastome of *S. dulcis* includes a small single-copy (SSC) region of 18,459 bp, a large single-copy (LSC) region of 89,353 bp, and two inverted repeats (IRs) regions of 27,222 bp. The total G/C content of *S. dulcis* is 37.7%. It shows that *S. dulcis* is closely related to *S. mombin* within Anacardiaceae. It will contribute to the conservation genetics of *S. dulcis* and the phylogenetic studies in Anacardiaceae.

## Introduction

*Spondias dulcis* Parkinson (1773) is a deciduous tree in the family Anacardiaceae (Buerki et al. 2010). The species originates in South America and is now widely cultivated in tropical areas (Buerki et al. 2010), partly because its fruits can be eaten. The species is called alien olive due to the fruits looking like olive. Here, we report the complete plastome of *Spondias dulcis*, which is expected to improve the quality of relevant collections and phylogenetic investigation of Anacardiaceae.

In this study, *S. dulcis* Parkinson was sampled from the city of Qionghai in Hainan, China (110.46°E,19.35°N). The tree of *Spondias dulcis* is 14 meters high and 16 cm in diameter at breast height. A voucher specimen (voucher code: D.-J. Chen, X.-R. Ke, A20, HUTB) and associated DNA were deposited in the Herbarium of Hainan University, Hainan province, China (code of herbarium: HUTB). The experiment was carried out as reported by Zhu et al. (2018). Cleaned sequencing data was assembled with GetOrganelle v1.7.5.0 (Santos and Almeida 2019). The plastome was annotated using the plastome of *Spondias bahiensis* P. Carvalho, Van den Berg and M. Machado (NC_030526.1) as a reference using Geneious v2021.1.1 (Biomatters Ltd, Auckland, New Zealand).

In this study, we find that the length of the complete plastome of *S. dulcis* is 162,256 bp. It includes 35 tRNA genes and eight rRNA genes, 86 protein-coding genes, and totally 130 genes. The complete plastome of *Spondias dulcis* includes a small single-copy (SSC) region of 18,459 bp, a large single-copy (LSC) region of 89,353 bp and two inverted repeats (IRs) regions of 27,222 bp. Eighteen protein-coding genes are duplicated in the IR. Seven tRNA are duplicated in the IR. 5S rRNA, 16S rRNA, 4.5S rRNA, and 23S rRNA are duplicated in the IR. The overall G/C content in the plastome is 37.7%, of which the corresponding value for the LSC, SSC, and IR regions are 35.7%, 32.2%, and 42.7%, respectively.

**Figure 1.**
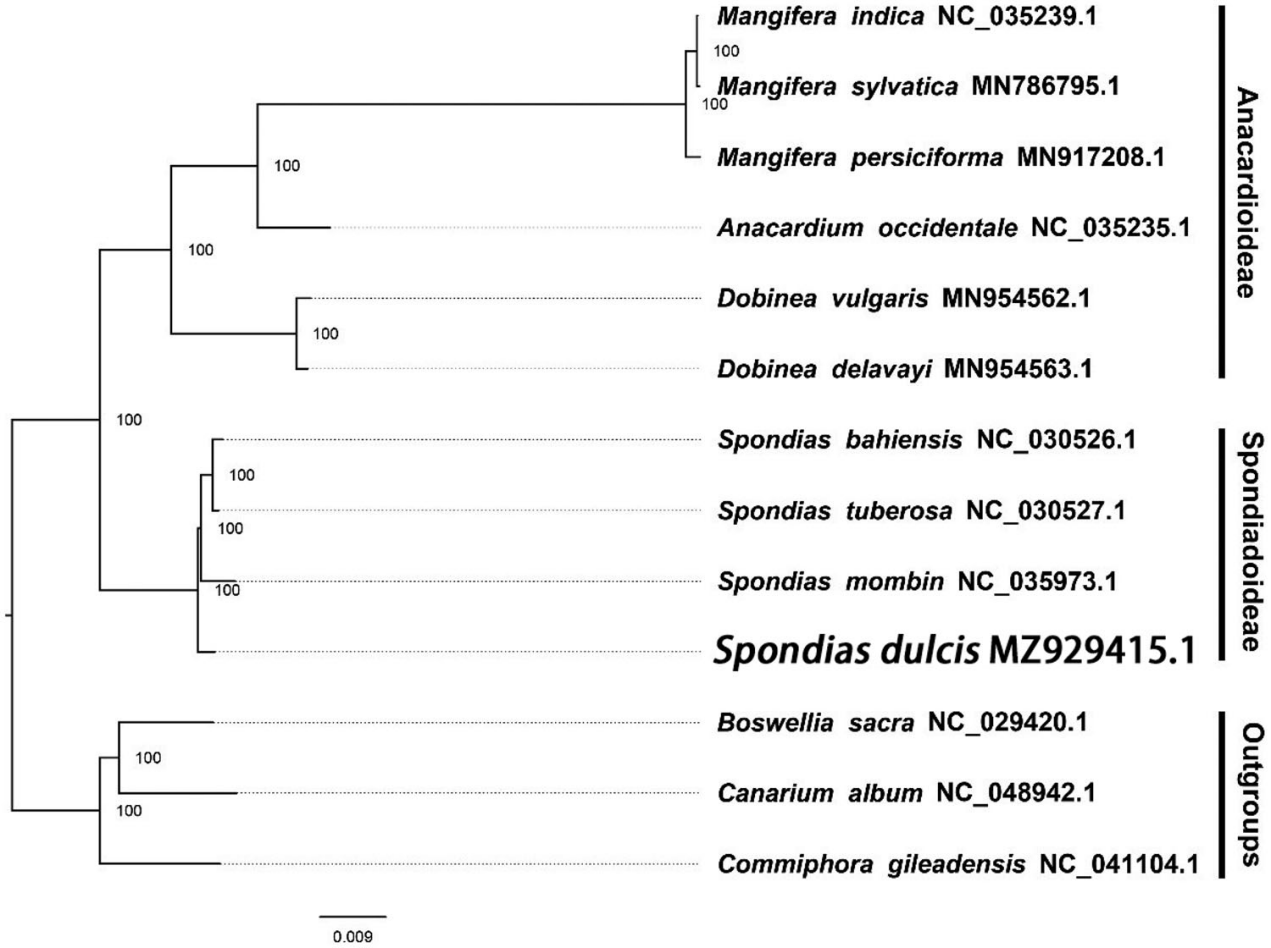
The maximum likelihood phylogeny obtained from 13 complete plastid sequences by RAxML. Support values are shown with bootstrap values at the nodes.

Based on existing data of related taxa from NCBI, we reconstructed a phylogenetic tree with RAxML with 1000 bootstraps with CIPRES (http://www.phylo.org/portal2/login!input.action). We found that *Spondias dulcis* is more closely related to *Spondias mombin* than other species in this genus in this study (Figure 1). Most nodes in the plastome maximum-likelihood (ML) tree of Anacardiaceae were highly supported. Furthermore, the plastid sequence of *S. dulcis* will promote relevant conservation and phylogenetic investigation of Anacardiaceae. Notably, Spondiadoideae was not supported as monophyletic as reported in previous studies (e.g. Pell et al. 2011; Weeks et al. 2014; Sun et al. 2016). Chen et al. (2016) found that *Spondias, Dracontomelon,* and *Buchanania* are in the same clade and sister to the rest of the family with moderate support, however, Muellner-Riehl et al. (2016) found that *Spondias* is closer to *Dracontomelon* than other genera within Anacardiaceae. Therefore, our study would bring great benefits to deepen the understanding complex relationships of plants such as those in Anacardiaceae.

## Data Availability

The results of the genome sequence data are publicly available in NCBI GenBank (https://www.ncbi.nlm.nih.gov/) with registration number MZ929415.1. The associated BioProject, SRA and Bio-Sample numbers are PRJNA748537, SRR15651129 and SAMN20858429 respectively. A specimen was deposited at Hainan University (https://ha.hainanu.edu.cn/home2020/) under the voucher number D.-J. Chen, X.-R. Ke, A20.
